# Inflammation and kidney involvement in human viral diseases caused by SARS-CoV-2, HIV, HCV and HBV

**DOI:** 10.1590/1678-9199-JVATITD-2020-0154

**Published:** 2021-07-28

**Authors:** Gustavo Ferreira da Mata, Danilo Euclides Fernandes, Eduardo de Paiva Luciano, Gabriel Teixeira Montezuma Sales, Michelle Tiveron Passos Riguetti, Gianna Mastroianni Kirsztajn

**Affiliations:** 1 Department of Medicine (Nephrology), Federal University of São Paulo (Unifesp), São Paulo, SP, Brazil.

**Keywords:** COVID-19, Glomerulonephritis, Hepatitis B, Hepatitis C, HIV, Inflammasome, SARS-COV-2

## Abstract

Inflammation is closely related to renal diseases. This is particularly true for renal diseases caused by infections as in viral diseases. In this review, we highlight the inflammatory mechanisms that underlie kidney dysfunction in SARS-CoV-2, human immunodeficiency (HIV), hepatitis C (HCV), and hepatitis B (HBV) infections. The pathophysiology of renal involvement in COVID-19 is complex, but kidney damage is frequent, and the prognosis is worse when it happens. Virus-like particles were demonstrated mostly in renal tubular epithelial cells and podocytes, which suggest that SARS-CoV-2 directly affects the kidneys. SARS-CoV-2 uses the angiotensin-converting enzyme 2 receptor, which is found in endothelial cells, to infect the human host cells. Critical patients with SARS-CoV-2-associated acute kidney injury (AKI) show an increase in inflammatory cytokines (IL-1β, IL-8, IFN-γ, TNF-α), known as cytokine storm that favors renal dysfunction by causing intrarenal inflammation, increased vascular permeability, volume depletion, thromboembolic events in microvasculature and persistent local inflammation. Besides AKI, SARS-CoV-2 can also cause glomerular disease, as other viral infections such as in HIV, HBV and HCV. HIV-infected patients present chronic inflammation that can lead to a number of renal diseases. Proinflammatory cytokines and TNF-induced apoptosis are some of the underlying mechanisms that may explain the virus-induced renal diseases that are here reviewed.

## Background

Renal diseases and inflammation are closely linked. This is particularly true for renal diseases caused by infections as in viral diseases. For example, the terms “glomerulonephritis” and “tubulointerstitial nephritis” themselves contain a suffix that means inflammation (from the Greek, ‘*itis*’).

In renal diseases, inflammation can be seen as complex interactions between renal parenchymal and resident immune cells (macrophages and dendritic cells) in association with the recruitment of circulating cells (monocytes, lymphocytes, and neutrophils), which stimulate receptors (Toll-like receptor and Nod-like receptor) that initiate major innate immunity pathways. One of them - the NLRP3 (or NACHT, LRR, and PYD domain-containing protein 3) inflammasome - induces inflammatory mediators secretion that ends in irreversible tissue damage and functional loss [1].

The podocytes, resident visceral epithelial cells that constitute the glomerulus, can express all the inflammasome components of NLRP3 that seems to be involved in the inflammatory mechanisms that underlies acute kidney injury (AKI) and chronic kidney disease (CKD) [2,3]. 

Regardless of the cause of CKD, fibrosis represents a common final pathway that leads to end-stage kidney disease (ESKD). Inflammation is involved in this process and transforming growth factor-β (TGF-β), which plays a potent anti-inflammatory effect by regulating negatively the renal inflammation, is an important mediator of renal fibrosis [4].

The NLRP3 inflammasome plays different roles in the pathogenesis of renal fibrosis as it mediates the inflammatory response and it also promotes pyroptosis, mitochondrial regulation, and myofibroblast differentiation [3]. NLRP3 activation in renal diseases aggravates inflammation, as well as the consequent fibrosis.

It is now known that NLRP3 has several inflammasome-dependent and independent functions in the kidney. The inflammasome-dependent NLRP3 mediates the progression of kidney diseases by escalating local kidney inflammatory response and promoting a crosstalk between the immune cells and the nonimmune renal cells. On the other hand, the inflammasome-independent NLRP3 regulates tubular epithelial cells apoptosis [3]. 

In this review, we discuss evidence on inflammatory processes that underlie some of the most frequent viral-induced kidney diseases - SARS-CoV-2, human immunodeficiency (HIV), hepatitis B (HBV), and C (HCV) viruses.

## Methods

**Figure 1.**
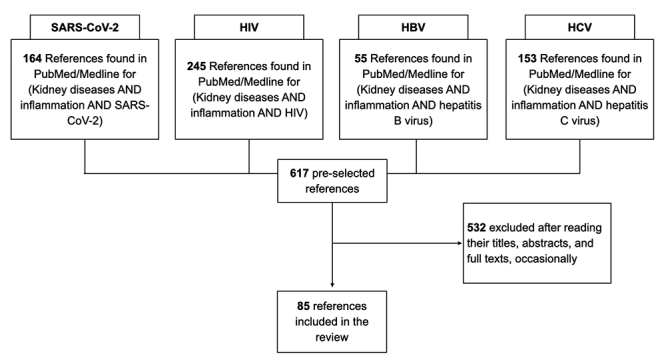
Article selection flow chart. HIV: human immune deficiency virus; HBV: hepatitis B virus; HCV: hepatitis C virus.

We used different strategies to search the subjects we present in this article, which included the PubMed/Medline database. We pre-selected four major topics based on their current relevance worldwide - infections due to SARS-CoV-2, HIV, HBV and HCV. The search strategy we used in PubMed was based on the terms described in Figure 1 that shows the article selection flow chart. We also included some relevant manuscripts that could enrich this review. 

### SARS-CoV-2

In December 2019, a group of patients with pneumonia of an unknown cause was observed in Wuhan, China [5]. On December 31, 2019, the Chinese Center for Disease Control and Prevention conducted epidemiologic and etiologic investigations and, then, the Chinese authorities reported to the World Health Organization (WHO) about such disease [5,6]. Chinese scientists identified a new viral agent - the novel coronavirus, at that time - on January 7, 2020. Few weeks later, on January 30, 2020, the WHO declared a “public health emergency of international concern” [6]. On February 11, 2020, the WHO officially named the disease as the novel coronavirus disease 2019 (COVID-19) and the Coronavirus Study Group (CSG) of the International Committee proposed to name the new coronavirus as SARS-CoV-2 [7]. 

Human coronaviruses are enveloped single-stranded RNA viruses, order *Nidovirales*. Seven different serotypes of coronaviruses cause human diseases, some of which lead to mild upper respiratory symptoms, but others may be fatal. SARS-CoV-2 is a betacoronavirus lineage B known to cause a severe respiratory disease that can affect other organs and tissues [8]. The COVID-19 raises at an overwhelming rate, reaching more than 32 million cases and more than 980,000 deaths worldwide [9]. In Brazil, there is more than 4,6 million cases and more than 139.000 deaths by September 2020 [9] and these numbers are still increasing.

To date, the COVID-19 does not has predilection for ethnicity and most patients (87%) were between 30 and 79 years old in China [6]. Recently, Kawasaki-like disease was reported in children, but further studies should confirm its association with the SARS-COV-2 infection [10]. Some factors seem to favor a more severe disease: hypertension, diabetes, obesity, aging, immunosuppression, among others [11].

The COVID-19 presents with a wide spectrum of clinical manifestations, from asymptomatic and oligosymptomatic to critical illness that requires intensive care and mechanical ventilation. The COVID-19 can evolve in three patterns: mild disease (80-85% of all cases) with fever, cough, sore throat, fatigue, malaise, headache, myalgia, and no evidence of pneumonia. In general, these symptoms are self-limited with no necessity of hospitalization or medical interventions. Despite the mild symptoms, the viral load can be high. The second pattern (10-15% of all cases) corresponds to a moderate-to-severe disease or a biphasic disease in which lungs are involved (pneumonia) besides the mild symptoms that can last for two weeks. The third pattern corresponds to the critical patients who present with shock, sepsis, severe hypoxemia, multiple organ failure, necessity of mechanical ventilation, and intensive care. About 5% of all cases may have this course, which symptoms can last for three weeks. Such critical cases initiate with high viral load followed by immune system overactivation when the viral load decreases [12]. Even though the COVID-19 is a respiratory disease, there is systemic involvement that commonly affects skin, intestinal tract, kidney, blood vessels, brain, and heart [13-17].

Laboratory findings reflect the changes in inflammatory biomarkers (C-reactive protein, interleukin [IL]-6, erythrocyte sedimentation rate, lactic dehydrogenase), coagulation disorders (thrombocytopenia and increased d-dimer), and lymphopenia, none of which are COVID-19 specific [14].

Renal involvement in the COVID-19 remains diverse and frequent, suggests a severe course and predicts a poor survival rate [18,19]. The renal disorders may present with hematuria and proteinuria (asymptomatic urinary abnormalities), collapsing glomerulopathy, acute diffuse glomerulonephritis, AKI (e.g. acute tubulointerstitial nephritis), endothelial injury and renal microvessel thrombosis [20-24].

The prevalence of AKI in patients varies from 3.0 to 30% [20,21,25,26], but it was also shown that the rate of AKI was 89.7% among the patients who needed mechanical ventilation compared with 21.7% among the other patients who did not need intensive care [27]. 

The kidneys receive a large amount of blood and they have a huge vascular network with a highly active endothelium. The SARS-CoV-2 gains access to the kidney through the bloodstream, as approximately 15% of the patients were found to have RNAemia [28]. Therefore, it becomes an active target for countless cytokines, complement system, and inflammatory processes. In addition, the kidneys express the angiotensin-converting enzyme 2 (ACE2) in large quantities, which SARS-CoV-2 uses to enter the host cells. ACE2 is mostly expressed on the apical brush-border membrane of the proximal tubule cells, but it is also present in podocytes at lower levels [29]. 

The critical patients that present with SARS-CoV-2- associated AKI show an increase in inflammatory cytokines such as IL-1β, IL-8, IFN-γ, TNF-α, when compared to the non-critical patients [8]. Besides the viral cytotoxic effect and the cytokines storm, a favorable environment for renal dysfunction includes intrarenal inflammation, increased vascular permeability, volume depletion, thromboembolic events in microvasculature and a persistent localized inflammatory process [8]. 

The pathophysiology of renal impairment in the COVID-19 is complex [27]. Some authors demonstrated that SARS-CoV-2 could directly infect human kidney tubules and induce cytoplasmic renal tubular inclusions [22,30]. They also found C5b-9 in renal tubular cells that may indicate complement system activation in SARS-CoV-2 infection [26]. An interaction among angiotensin II (AngII) overactivity, innate/adaptive immune response, and complement and coagulation systems could influence AKI severity and its outcomes. Inflammation-induced erythrocyte aggregation and heme-mediated injury may increase oxidative stress, inflammation, and complement activation, which may promote microvascular injury [29].

In addition to the inflammatory/immune-mediated mechanisms the SARS-CoV-2-associated AKI may be ascribed to different causes such as impaired gas exchange, hemodynamic alterations including right heart failure, fluid overload, and systemic congestion, injurious mechanic ventilation strategies, and secondary infections/sepsis [20].

In general, viral infections can cause many kidney diseases, which diagnosis depend on clinical and laboratory investigations, as well as histological analysis. Many mechanisms are involved, including viral kidney tropism, induction of abnormal immune complexes, direct cytopathic effects, and multiorgan failure [31]. SARS-CoV-2 infection can trigger glomerular diseases (several types were reported, including collapsing glomerulopathy) or aggravate a pre-existing disease [23,32].

Besides tubular or glomerular disorders, there is endothelial dysfunction and microvascular injury [33]. *Post mortem* analyses indicate that kidney and lung endothelium are affected, which may be responsible for proteinuria [19] and prothrombotic state [33]. As previously mentioned, SARS-CoV-2 uses the ACE2, which is expressed by the endothelial cells, to infect the host cells [34]. There is evidence of direct viral infection of such cells and diffuse endothelial inflammation. The endothelial involvement could explain the variety of clinical manifestations in different organs and systems. Despite the progress towards COVID-19 understanding, further studies will clarify the underlying mechanisms through which SARS-CoV-2 affects the kidneys and, then, more effective approaches may be suggested.

### HIV

Human immunodeficiency virus (HIV) is an RNA virus. Since the first reports in the 1980s, natural history of HIV infection has changed drastically due to the advent of antiretroviral therapy (ART). Currently, the life expectancy of HIV-infected individuals who are under ART matches the life expectancy of those who are HIV-uninfected [35]. 

A pro-inflammatory state was described since the beginning of the HIV pandemic, which includes higher serum levels of IL-6, TNF-α, TGF-β, and other inflammatory molecules [36]. Additionally to the immune system aging (known as “inflamm-aging”), those who live with HIV becomes more vulnerable to a chronic inflammatory state that can lead to some diseases [37], including renal dysfunction.

With regards to kidneys, we can schematically divide those disorders into four main groups, all of which can cause CKD or ESKD: (1) AKI; (2) HIV-associated kidney disease; (3) associations with other risk factors for CKD; and (4) treatment-related toxicity [38].

AKI remains very common among HIV-infected individuals and accounts for the increase in the incidence of renal replacement therapy [38-40]. Besides age-related diseases such as diabetes, hypertension, CKD, and liver diseases, sepsis still is the pivotal risk factor for AKI [38,40,41].

Three major scenarios are more likely in HIV-associated kidney disease: HIV-associated nephropathy (HIVAN), HIV-associated immune complex kidney disease (HIVICK), and thrombotic microangiopathy. HIVAN usually happens in the late stages of HIV infection with or without acquired immunodeficiency syndrome (AIDS). Some *APOL1* polymorphisms may suggest a higher risk for HIVAN [38,42]. Management of HIVICK remains unclear [38], as the pathophysiology of thrombotic microangiopathy in HIV [43], but some studies have shown that HIV is highly associated with thrombotic thrombocytopenic purpura [44-46] or, less frequently, hemolytic uremic syndrome [47].

The associations between HIV infection and CKD itself or its risk factors include both modifiable factors (diabetes, hypertension, and hepatitis B and C) and non-modifiable ones (ethnicity) [38,48,49]. Although 5.8% of acute HIV-infected subjects may present proteinuria, the prevalence of estimated glomerular filtration rate (eGFR) < 60 mL/min tends to be even lower (0.5%) among those individuals [38,50-52]. Over the past decades and in light of modern antiretroviral regimens, focal segmental glomerulosclerosis, diabetic nephropathy, arteriolar nephrosclerosis (with or without hypertension), immune complexes diseases (with or without liver disease), and toxicity replaced the previous most frequent histopathological finding, the collapsing glomerulopathy [38].

Treatment-related toxicity usually happens after long-term exposure to nephrotoxic antiretrovirals such as tenofovir (TFV), which is frequently used for treating and preventing (pre- and post-exposure prophylaxis) HIV infection. Other antiretrovirals have been associated with nephrotoxic events as indinavir, ritonavir, atazanavir, and lopinavir, but only TFV nephrotoxicity is well-described. TFV commonly causes isolated proximal tubular dysfunction by intoxicating the mitochondria within the tubular epithelial cells, which can lead them to apoptosis. Laboratory findings can show low molecular weight proteinuria, mild albuminuria, a decrease in eGFR, and Fanconi syndrome. The main risk factors for such effects are eGFR < 60 mL/min and ART with TFV boosters (protease inhibitors, such as atazanavir and ritonavir) that plays an important combination for treating HIV-infection. TFV-nephrotoxicity can be reverted after interrupting its use, although some reports suggest irreversibility [38,53]. 

### HBV

HBV infection is of global public health concern. In 2015, the WHO estimated 257 million HBV-infected individuals around the world and approximately 887,000 people died from HBV-related liver disease by that year [54]. Nearly 250 to 350 million people (5% of the world’s population) are chronically HBV-infected, which makes HBV to be one of the most common human pathogens. Additionally, 3% to 5% of the patients with chronic HBV infection may develop kidney disease as a complication [55].

The HBV-associated renal diseases frequently occur during the childhood in endemic areas, which likely turn the patient into a chronic carrier [56]. Children with HBV-related membranous nephropathy (MN) have a high spontaneous remission rate and rarely progress to ESKD. Otherwise, adults with HBV-related MN show higher progression risk [55].

Among the glomerular diseases most associated with HBV infection are MN, membranoproliferative glomerulonephritis (MPGN), IgA nephropathy (IgAN), cryoglobulinemia, and polyarteritis nodosa (PAN; Kussmaul-Meier disease). About 10% of the HBV-infected subjects are coinfected with HIV and 10-30% are coinfected with HCV. As coinfection with HIV and HCV is quite common, those at higher risk (e.g., intravenous drug use, unprotected sex) should also be screened for these viruses [57]. Another reason for screening the patients with proteinuric glomerular diseases for HBV infection is that immunosuppressive therapy can exacerbate HBV replication. 

The diagnosis of HBV-related glomerulonephritis requires a meticulous laboratory investigation, which includes serum biomarkers levels, detection of viral particles in blood sample, and detection of HBV antigens in kidney histopathological study. Furthermore, any other causes of glomerular disease must be ruled out. HBV infection can be silent and can show no sign of liver damage - as increased hepatic enzymes -, thus a liver biopsy can clarify the level of organ injury [58].

HBV-associated secondary MN, as other forms of MN, usually presents with proteinuria, which can reach a nephrotic range. Compared with the patients who have primary MN, those with HBV-associated MN are more likely to have microscopic hematuria and lower complement levels. At the histopathological study, the presence of mesangial or subendothelial immune deposits associated with its typical subepithelial localization may suggest secondary rather than primary MN [59]. It was shown a low prevalence of anti-phospholipase A2 receptor (PLA2R) antibodies and/or PLA2R staining of the immune deposits in HBV-associated MN [60,61].

HBV-associated MPGN, as other forms of MPGN, presents with hematuria, variable degrees of proteinuria, reduced GFR, and hypertension. The deposition of circulating antigen-antibody complexes in the mesangium and subendothelial space is observed in HBV-associated MPGN. Both HBsAg and HBeAg deposition have been implicated in this disorder, although their exact role remains uncertain. Compared to HCV, HBV infection is a rare cause of mixed cryoglobulinemia, which can be associated with MPGN [62].

PAN is a necrotizing vasculitis that affects both small- and medium-sized blood vessels, which involves multiple organs frequently. The PAN-related renal involvement leads to variable degrees of reduced glomerular filtration ratio (GFR) and hypertension. The clinical features of HBV-associated PAN are similar to the idiopathic one. HBV-associated PAN typically occurs within four months after the onset of HBV infection. The vessel wall deposits of circulating antigen-antibody immune complexes triggers the following inflammatory processes [56].

As concerned to other underlying mechanisms of HBV-related renal diseases, the presence of HBV-DNA and RNA in renal tubular epithelial cells (RTECs) suggests direct virus-induced injury. Increased proinflammatory cytokines are also observed under those conditions. Apoptosis, which is mostly induced by the TNF-related apoptosis-inducing ligand (TRAIL), plays a significant role in the pathogenesis of HBV-infections. However, the effects of HBV X protein (HBx) on TRAIL-induced apoptosis of RTECs, especially under certain inflammatory conditions remain unclear. HBx synergizes with proinflammatory cytokines to significantly increase TRAIL-induced apoptosis of RTECs. HBx markedly upregulates the death receptor-4 (DR4) expression by enhancing the activation of nuclear factor-kappa B when proinflammatory cytokines are present. A dramatic increase in DR4 expression leads to the sensitization of RTECs to TRAIL-induced apoptosis. Furthermore, in those with HBV-associated glomerulonephritis, DR4 expression in the kidneys is significantly elevated and it correlates positively with HBx and proinflammatory cytokines expression. Such findings provide a novel insight into the underlying mechanisms of renal tubule lesions induced by HBx in HBV-associated glomerulonephritis [63]. 

Notch1 plays an important part in regulating immune responses and epithelial-mesenchymal transdifferentiation (EMT). It was previously observed inflammatory cell infiltration and tubulointerstitial fibrosis in the renal biopsies from the patients with HBV-associated glomerulonephritis. It has been suggested that Notch1 is significantly associated with renal tubular and interstitial lesions. Notch1 can mediate HBx-induced EMT of human proximal tubular epithelial cells (HK-2), promote HBx-induced increases in immune molecule expression and exacerbation of cytokine disorders, which may contribute to the progression of HBV-associated glomerulonephritis. Inhibitors of Notch1 signaling may be useful as new therapeutic strategy at HBV-associated glomerulonephritis [64].

### HCV

HCV is an RNA virus from the *Flaviviridae* family which was first described in 1989. Prior to the HCV description, many cases of chronic hepatitis among individuals who underwent blood transfusion history were called non-A, non-B hepatitis, since no etiology could be identified, as well as most cases of cryoglobulinemia were considered idiopathic [65]. Nowadays, HCV is an endemic infection worldwide, with more than 170 million people infected that can complicate with cirrhosis, hepatocellular carcinoma and cryoglobulinemia [66]. Recently, the direct-acting antivirals revolutionized the treatment of HCV as the sustained viral response jumped from close to 50% to over 90% [67]. Even more striking improvement is found among poor responders, which usually include CKD and cirrhotic patients [68]. These results are the motivation to 2030 WHO’s goal to reduce the incidence of HCV in 80% and mortality in 65% [69].

HCV has mainly hepatocytes tropism but it can also infect lymphocytes, which may explain the extrahepatic manifestations, as some cohorts show a prevalence close to 70% in chronic infected patients, including porphyria cutanea tarda, insulin resistance, thyroiditis, lymphoma and systemic vasculitis [70,71]. Clinical manifestations related to the kidneys are commonly seen, as hematuria, proteinuria, AKI, edema and hypertension, because of which annual screening with eGFR and urinalysis is recommended in HCV-infected patients [72]. Mcguire *et al*. have demonstrated through protocol kidney biopsies during liver transplantation that up to 85% of chronic infected patients present any kidney involvement that can be attributed to HCV [73]. Immune complex-mediated MPGN associated to cryoglobulinemia remains the most common renal disease, usually manifested as a systemic vasculitis - purpura, arthralgia, peripheric neuropathy and nephropathy - characterized by subendothelial deposits of immune-complexes containing immunoglobulin M (IgM) autoantibodies against the Fc fragment of immunoglobulin G (IgG) that precipitate in cold, called cryoglobulins [74]. 

Cryoglobulinemia is classified in three types according to autoantibodies clonality. Type 1 is associated with lymphoproliferative diseases and both IgG and IgM are monoclonal. Type 2, or mixed cryoglobulinemia, has monoclonal IgM and polyclonal IgG, while type 3 is completely polyclonal. The last two are associated with autoimmune diseases, and especially with HCV chronic infections, with a prevalence of around 90% among those with mixed cryoglobulinemia, including evidence of viral-containing immune complexes [74,75]. This phenomenon can be explained by the HCV ability to infect B lymphocytes through the CD81 glycoprotein, as well as by the mimicry of HCV-specific proteins, as NS3, which is similar to the IgG-Fc component, stimulating autoantibodies production [73,76]. Besides that, an impairment in phagocyte system activity has been correlated with a higher renal exposition to cryoglobulins, which can explain why up to 50% of the HCV-infected patients present serum cryoglobulins but only around 5% of them develop active inflammatory disease [77,78]. Another mechanism involved in the HCV-related immunological dysregulation is increased levels of B lymphocyte stimulator, a cytokine related to B cell proliferation and survival [79,80]. 

Kidney histology in cryoglobulinemia is usually characterized by endocapillary and mesangial proliferation, with a classical double-contoured appearance, a pattern known as MPGN. Sometimes hyaline intracapillary thrombi, microtubular structures and fibrillar deposits can also be observed by electron microscopy [75]. Other kidney disorders less frequently associated with HCV are immune-complex-related glomerular diseases as MPGN without cryoglobulinemia, MN and fibrillary glomerulopathy. Mesangial proliferative glomerulonephritis, tubulointerstitial nephritis, PAN and focal segmental glomerulosclerosis can also be found and for the last two a direct virus-induced damage to podocytes and tubular cells hypothesis has been defended [68,67]. 

Despite the limitations of the immunosuppressive therapy and considering that the main goal is to reach a sustained viral response, it is recommended to perform kidney biopsy when there is clinical suspicion of glomerulopathies - proteinuria or progressive kidney failure associated with hematuria [72]. 

As already mentioned, the treatment of HCV-associated nephropathy invariably needs antiviral therapies. Some cases that present with a more severe kidney involvement, such as nephrotic syndrome or AKI, should also be treated with immunosuppressive agents. Rituximab or cyclophosphamide plus corticosteroids remain the most common associations. Those who present with a life-threatening condition may benefit from therapeutic plasma exchange [81]. Since 2014, when direct acting antivirals were first described - NS3/4A, NS5A and NS5B inhibitors - the treatment of HCV has turned highly effective, and some combinations can reach 100% of sustained viral response. Unfortunately, although these new drugs can eliminate HCV, immune manifestations such as glomerulonephritis may still remain with an unsatisfactory remission rate, which emphasizes the need for considering immunosuppressive therapy in critical cases [68]. An HCV-induced immunological trigger may be responsible for a poor response to the treatment because HCV can infect B lymphocytes in a partially irreversible way, which may explain why many patients benefit from the immunosuppressive therapy [67].

## Conclusion

Inflammation participates in the pathogenesis of renal diseases, with a relevant role in such affections associated with infections. Moreover, it influences progression to ESKD.

In 2020, the outbreak of a new viral disease, the COVID-19, drew attention to the inflammatory mechanisms that underlies this and other viral infections. In COVID-19, inflammation was associated with a myriad of clinical manifestations, and severe renal involvement. The unique features of SARS-CoV-2 related to renal diseases and their inflammatory mechanisms were described in the present study, as those of the HIV, HCV and HBV infections. 

### Abbreviations

ACE2: angiotensin-converting enzyme 2; AIDS: acquired immunodeficiency syndrome; AKI: acute kidney injury; AngII: angiotensin II; APOL1: apoliprotein 1; ART: antiretroviral therapy; CKD: chronic kidney disease; COVID-19: coronavirus disease 2019; DR4: death receptor-4; eGFR: estimated glomerular filtration rate; EMT: epithelial-mesenchymal transdifferentiation; ESKD: end-stage kidney disease; GFR: glomerular filtration rate; HBsAg: hepatitis B surface antigen; HBV: hepatitis B virus; HCV: hepatitis C virus; HIV: human immunodeficiency virus; HIVAN: HIV-associated nephropathy; HIVICK: HIV-associated immune complex kidney disease; IgAN: IgA nephropathy; IgG: immunoglobulin G; IgM: immunoglobulin M; IL: interleukin; INF: interferon; MN: membranous nephropathy; MPGN: membranoproliferative glomerulonephritis; NLRP3: NACHT, LRR, and PYD domain-containing protein 3; PAN: polyarteritis nodosa; PLA2R: phospholipase A2 receptor; RTECs: renal tubular epithelial cells; SARS-CoV-2: severe acute respiratory syndrome coronavirus 2; TFV: tenofovir; TGF: transforming growth factor; TNF: tumor necrosis factor; TRAIL: TNF-related apoptosis-inducing ligand; WHO: World Health Organization. 
